# Mobile Exercise Apps and Increased Leisure Time Exercise Activity: A Moderated Mediation Analysis of the Role of Self-Efficacy and Barriers

**DOI:** 10.2196/jmir.4142

**Published:** 2015-08-14

**Authors:** Leib Litman, Zohn Rosen, David Spierer, Sarah Weinberger-Litman, Akiva Goldschein, Jonathan Robinson

**Affiliations:** ^1^ Lander College Psychology Department Kew Gardens Hills, NY, NY United States; ^2^ Mailman School of Public Health GRAPH Center Columbia University New York, NY United States; ^3^ Long Island University Division of Athletic Training, Health and Exercise Science Brooklyn, NY United States; ^4^ Marymount Manhattan College Psychology Department New York, NY United States; ^5^ Lander College Computer Science Department Kew Gardens Hills, NY, NY United States

**Keywords:** mobile health, apps, exercise, barriers to exercise, self-efficacy, BMI

## Abstract

**Background:**

There are currently over 1000 exercise apps for mobile devices on the market. These apps employ a range of features, from tracking exercise activity to providing motivational messages. However, virtually nothing is known about whether exercise apps improve exercise levels and health outcomes and, if so, the mechanisms of these effects.

**Objective:**

Our aim was to examine whether the use of exercise apps is associated with increased levels of exercise and improved health outcomes. We also develop a framework within which to understand how exercise apps may affect health and test multiple models of possible mechanisms of action and boundary conditions of these relationships. Within this framework, app use may increase physical activity by influencing variables such as self-efficacy and may help to overcome exercise barriers, leading to improved health outcomes such as lower body mass index (BMI).

**Methods:**

In this study, 726 participants with one of three backgrounds were surveyed about their use of exercise apps and health: (1) those who never used exercise apps, (2) those who used exercise apps but discontinued use, and (3) those who are currently using exercise apps. Participants were asked about their long-term levels of exercise and about their levels of exercise during the previous week with the International Physical Activity Questionnaire (IPAQ).

**Results:**

Nearly three-quarters of current app users reported being more active compared to under half of non-users and past users. The IPAQ showed that current users had higher total leisure time metabolic equivalent of task (MET) expenditures (1169 METs), including walking and vigorous exercise, compared to those who stopped using their apps (612 METs) or who never used apps (577 METs). Importantly, physical activity levels in domains other than leisure time activity were similar across the groups. The results also showed that current users had lower BMI (25.16) than past users (26.8) and non-users (26.9) and that this association was mediated by exercise levels and self-efficacy. That relationship was also moderated by perceived barriers to exercise. Multiple serial mediation models were tested, which revealed that the association between app use and BMI is mediated by increased self-efficacy and increased exercise.

**Conclusions:**

Exercise app users are more likely to exercise during their leisure time, compared to those who do not use exercise apps, essentially fulfilling the role that many of these apps were designed to accomplish. Data also suggest that one way that exercise apps may increase exercise levels and health outcomes such as BMI is by making it easier for users to overcome barriers to exercise, leading to increased self-efficacy. We discuss ways of improving the effectiveness of apps by incorporating theory-driven approaches. We conclude that exercise apps can be viewed as intervention delivery systems consisting of features that help users overcome specific barriers.

## Introduction

### Background

In recent years, the mobile apps market has seen a proliferation of medical and health apps [1-5]—apps whose purpose is to promote health and improve the delivery of health care. Of the wide variety of mobile health apps, exercise and fitness apps are the most popular, accounting for 39% of health-related apps [6].

Lack of exercise puts individuals at an increased risk of numerous chronic health conditions, including metabolic syndrome, cancer, depression, and osteoporosis [7]. It is well documented that sufficient exercise is critical for the promotion of long-term health, weight management, and improved management and prognosis of a variety of chronic illnesses, including a 35% reduction in cardiovascular mortality and a 33% reduction in all-cause mortality [8]. Despite the importance of physical activity, an alarming 30% of people throughout the world are physically inactive [9].

Because of their widespread use, exercise apps have the potential to dramatically improve health outcomes in the United States and around the world, and may thus play an increasingly important role in the public health effort to increase population-wide exercise levels. Empirical evidence is beginning to emerge that the use of exercise-related mobile health technology may be associated with increased exercise levels [10-18] (see [19] for review), and such devices are also beginning to be used in clinical settings [20]. However, the mechanisms by which exercise apps may impact behavior change and health outcomes have not been thoroughly explored.

### Current Research

Exercise apps can be viewed as collections of features, each of which has the potential to target specific aspects of cognition, affect, and behavior. Features of exercise apps include providing feedback based on tracking user exercise activity, providing motivational messages, demonstrating the right way to exercise, setting and monitoring goals, incorporating social media, and helping users schedule their exercise program. There are currently over 1000 exercise apps for mobile devices on the market incorporating a myriad of combinations of specific features [6,21]. In this study, we explore how apps may impact behavior at a broad level, by focusing on two variables that are key predictors of physical activity: self-efficacy and barriers to exercise. Most broadly, app features may be conceptualized as targeting specific barriers. To the extent that an app may help an individual overcome specific barriers, it may be expected to increase self-efficacy, leading to increased exercise and, over time, improved health outcomes.

In this study, we test various models of how these variables may be influenced by app use and in turn may help promote increased levels of exercise and health. The models’ starting point is the well-established link between exercise and health outcomes. We use body mass index (BMI) as an indicator of health because of its known association with overall health outcomes [22] and its association with exercise levels [23]. We expect to replicate this latter association in this study, in that participants in our sample who report exercising more regularly are expected to have an overall lower BMI. Having replicated that link, we will explore ways that using an exercise app may be associated with changes in BMI, through the effects that app use has on increasing exercise. Specifically, we will test the hypothesis that the use of exercise apps will be associated with greater levels of exercise, which in turn will be associated with lower BMI.

**Figure 1:**
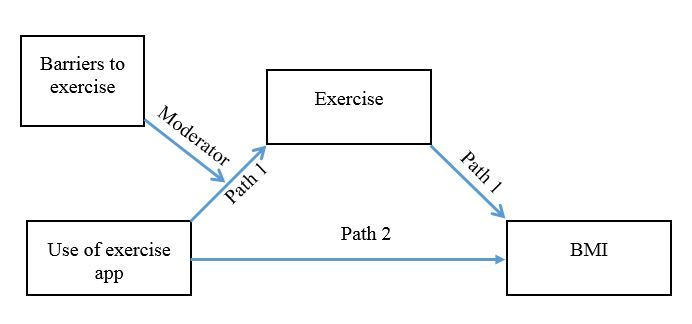
Model of relations between app use, exercise, and BMI, with barriers to exercise as the moderator.

We further predict that individuals who have a high number of barriers to exercise will exercise more when they use an exercise app compared to those who do not use an app. However, individuals who have relatively few barriers will be less likely to benefit from using an exercise-focused app. Figure 1 presents a model of relations between app use, barriers to exercise, exercise levels, and BMI. The model predicts that app use leads to increased exercise (Path 1) and that increased exercise leads to decreased BMI (Path 2). The model also predicts that the effect of app use on exercise (Path 1) will be moderated by barriers. In a separate model, we examine the effect of app use on self-efficacy. App users are expected to have higher self-efficacy, which will mediate exercise levels and in turn mediate BMI.

## Methods

### Participants

A total of 726 participants completed the study; 54.9% (399/726) were male. The dropout rate was 7%. Participants were recruited through Amazon’s Mechanical Turk platform. Each participant was paid US $2.50 for completing the study. Participants’ ages ranged between 18 and 74 (mean 32.4, SD 11.1).

### Design

The study was posted on Amazon Mechanical Turk (MTurk) [24] where a link was made available to a survey, hosted on the Qualtrics website. Participants were asked about their use of exercise apps, and those answers resulted in their assignment to one of three groups: (1) those who never used an exercise app, (2) those who used an exercise app in the past but had since stopped, and (3) those currently using an exercise app. Exercise apps were defined as follows: “Have you ever used an exercise application on any device (iPhone, iPad or Android)? An exercise application is a downloadable App for a mobile device. It is intended to help you exercise (note, apps that help with diet but not exercise are not considered exercise apps).”

The participants who had used an exercise app either currently or in the past were asked questions pertaining to their app use and to the app itself, such as how long they used the app for, if they found it helpful, and what features the app has (eg, social features such as the ability to post results on Facebook, feedback structure, ability to input data). They were also asked to choose their app from a list of apps. In addition, each participant was asked to fill out measures that assessed their level of physical activity, their perceived barriers to and perceived benefits from exercise, their exercise self-efficacy, and self-reported weight and height. The three groups were then compared to see if using an exercise app was associated with our key outcomes, including BMI, frequency of exercise, metabolic equivalent of task (MET) levels, self-efficacy, and barriers to exercise.

The study with all surveys and questions can be found in Multimedia Appendix 1.

### Measures

#### International Physical Activity Questionnaire

Physical activity (PA) was assessed using the International Physical Activity Questionnaire—Long Form (IPAQ) [25]. The IPAQ is a survey that consists of 27 questions on the frequency and duration of PA during the last 7 days. The survey covers five separate domains: (1) job-related physical activity, (2) transportation-related PA such as biking or walking, (3) PA related to housework and gardening, (4) PA done during leisure time, and (5) time spent sitting. Within these activity domains, levels of activity intensity were estimated and assigned an MET: moderate PA=4 MET, vigorous PA=8 MET, walking=3.3 MET, sitting=1 MET. The IPAQ was scored by multiplying the frequency (days per week), duration (minutes per day), and the intensity (MET) of the activities performed, resulting in a MET minutes per week score.

#### Exercise Benefits/Barriers Scale

The Exercise Benefits/Barriers Scale (EBBS) [26] consists of 43 items with two subscales: one relating to perceived benefits to exercise (29 items) and one relating to perceived barriers of exercise (14 items). The scale has an overall internal reliability of .95, test-retest reliability of .85 [27], and predicts physical activity levels [28].

#### Exercise Confidence Survey

The Exercise Confidence Survey (ECS) is a 12-item scale that measures exercise self-efficacy [29], using a 5-point Likert scale. The scale has an alpha reliability coefficient of .92, and it significantly predicts self-reported activity [30].

### Assessment of Long-Term Exercise Frequency

The IPAQ is limited to assessing activity during a 1-week interval. However, activity levels on any given week can be influenced by multiple factors such as illness, work and family commitments, holidays, and vacations. Therefore we included a question to assess longer-term exercise patterns that asked participants, “On average, how often have you exercised over the last several months?” The available response options were (1) I don’t currently exercise, (2) I am exercising but infrequently (a few times a month at most), (3) I am exercising about once a week, (4) I exercise 2 to 3 times a week, and (5) I exercise more than 3 times a week. Responses 4 and 5 were labeled as “active”.

### Statistical Analyses

#### Moderation and Mediation Models

Moderation and mediation models were computed utilizing PROCESS, a mediation and moderation software package [31]. The three app use groups were dummy coded [32], with non-users being the reference group against which current and past users were compared. Barriers to exercise were centered, and cross products of barriers to exercise and app use conditions were computed to produce the two interaction terms required to represent the interaction between the app use condition and barriers to exercise. Exercise frequency was then regressed onto the two contrast variables, with additional covariates included in the model being education level, age, and income.

#### Mediation

The three levels of app use were dummy coded as in the moderation analysis above. Each of the two dummy-coded variables was entered into the regression equation, with the other dummy-coded variable entered as a covariate, and age as an additional covariate in the model. To test this potential mediation effect, we followed the bootstrapping method, utilizing 5000 iterations [33]. The bootstrapping procedure tests the null hypothesis that the indirect path from the interaction term to the dependent variable via the mediator does not significantly differ from zero. If zero is not contained within the confidence intervals (CI) computed by the bootstrapping procedure, one can conclude that the indirect effect is indeed significantly different from zero at *P*<.05.

#### Moderated Mediation

Moderated mediation was modeled by entering the contrast between currently using and never used groups (contrast 1) as the predictor, barriers to exercise as the moderator, and BMI as the outcome variable. As described in [31], the contrast between past users and current users groups (contrast 2), the moderator, and the interaction between contrast 1 and the moderator, were entered as covariates. The index of moderated mediation [31] uses the bootstrapping method to test the null hypothesis that the indirect path from the interaction term to the dependent variable via the mediator does not significantly differ from zero.

### Descriptive Statistics

Significant age differences in app use were observed. Specifically, participants who were older than 35 years were more likely to have never used an exercise app. Because exercise levels, BMI, and other outcome variables in this study such as attitudes toward exercise and perceived barriers can all differ based on age, we used two separate approaches in controlling for age: (1) exclude participants older than 35 and (2) covarying out the effect of age.

For the purposes of presenting the results descriptively (and the corresponding chi-square tests of independence), we excluded participants older than 35. The purpose of these analyses is to establish the percentage of participants in each app use condition who exercise regularly. Thus, for these analyses, rather than covarying out age, we included only the participants who were younger than 36.

A total barriers score was computed by summing items across all of the barriers examined by the scale. For the purposes of presenting the results descriptively, we dichotomized the barriers scale into high and low barriers individuals, based on the mean of the total barriers score.

For mediation and moderation modeling, all participants, including those above 35, were included in the analyses, and age was entered as a covariate in all models.

## Results

### Demographic Characteristics of the Sample

Nearly two-thirds of participants (464/726, 63.8%) reported never having used an exercise app (non-users), 15.8% (115/726) reported having used an app in the past but stopping (past users), and 20.2% (147/726) reported using an exercise app currently (current users). Non-users (NU) were older on average (34.3 years old) than past users (PU) (29.1 years old) and current users (CU) (28.1 years old): *F*_2,721_=20.4, *P*<.001. Sheffe-corrected post-hoc comparisons showed that current and past users did not differ in age from each other (*P*>.9) but both were significantly younger than non-users (*P*s<.001). There were also differences in BMI between the three groups (NU=26.9, PU=26.7, CU=25.2), *F*_2,721_=4.3, *P*=.014.

### Internal Consistency of Assessment Measures

Table 1 presents zero-order correlations between measures used in this study, which reveal both expected and internally consistent patterns of associations. Participants who reported a lot of barriers to exercise were less active on all measures, had lower self-efficacy, and higher BMI. Self-efficacy was positively correlated with all measures of physical activity, and negatively correlated with BMI. BMI was negatively correlated with length of app use (in weeks) for the current users group (*r*_146_=-.19, *P*<.05), but not for the past users group (*r*_114_=.05, *P*=.55).

Exercise frequency (EF) was revealed to be correlated with activity levels as measured by the IPAQ (*r*_724_=.32 between EF and Total Leisure MET, *P*<.001), thus establishing the convergent validity of the long-term exercise frequency measure. People generally exercise during their leisure times. Thus, exercise levels should correlate strongly with vigorous leisure time activity. Consistent with this approach, exercise frequency was correlated with vigorous leisure MET levels and with total leisure MET levels. However, EF was less strongly correlated with moderate leisure MET levels, and not at all with work MET levels.

Exercise frequency was also correlated with barriers, self-efficacy, and BMI. Although both the exercise frequency and IPAQ measures were significantly correlated with self-efficacy, barriers, and BMI, exercise frequency was more strongly correlated with these measures than the IPAQ. This is likely due to the IPAQ’s exclusive focus on activities during a single week. Because exercise frequency appears to be a more reliable measure of long-term exercise patterns, we use exercise frequency as the outcome variable in our mediation and moderation models. Since the IPAQ can provide insight as to which areas of daily life are likely to be impacted by exercise apps, in subsequent analyses the IPAQ is used to supplement the exercise frequency measure of activity.

**Table 1:** Zero-order correlations for the total sample (zero-order correlations between all outcome measures, mediators, and moderators).

Measures^a^	1	2	3	4	5	6	7	8
Exercise frequency (1)	–	.32^b^	.34^b^	.22^b^	.01	-.51^b^	.53^b^	-.19^b^
Total leisure MET (2)		–	.93^c^	.65^b^	.14^c^	-.2^b^	.28^b^	.08^c^
Vigorous leisure MET (3)			–	.46^b^	.09	-.22^b^	.28^b^	-.1^c^
Moderate leisure MET (4)				–	.15^c^	-.1^c^	.18^b^	-.05
Work MET (5)					–	-.01	.19^c^	.07
Barriers scale (6)						–	-.53^b^	.27^b^
Self-efficacy scale (7)							–	-.14^b^
BMI (8)								–

^a^Numbers in parentheses correspond to column numbers.

^b^Indicates a significant correlation at the *P*<.001 level.

^c^Indicates a significant correlation at the *P*<.05 level.

### Exercise Differences Between App Use Conditions

In the analyses below, we examine whether the use of exercise apps is associated with increased activity levels. As discussed above, for this analysis only participants below age 35 were included. Thus, for the descriptive statistics presented in Tables 2 and 3, all participants were below age 35. These participants did not differ with respect to age: *F*_2,496_=1.6, *P*=.2.

In the first analysis, we use exercise frequency as the outcome variable. For this analysis, we considered individuals who reported exercising two or more times a week as active. Overall, current users were more likely to be active (73%) compared to non-users (45.8% active), and past users (46.1% active),*χ* ^2^=30.6, *P*≤.001. Past users and non-users were not different from each other (see Table 2).

**Table 2:** Percent of active participants for three app use groups across two levels of barriers.^a^

	Active app users, %			df	*X* ^2^	*P* value		
	Non-users	Past users	Current users			Current users vs non-users	Current users vs past users	Non-users vs past users
All subjects	45.8	46.1	73	2	30.6^b^	<.001	.0001	.53
High barriers	32.2	23.8	60	2	20.7^b^	.001	.001	.44
Low barriers	68.7	72	84.1	2	7.2^c^	.004	.142	.37

^a^Comparisons of current app users, those who began using an app and then stopped, and those who never used an app on self-reported frequency of exercise. Exercise at a rate of twice per week was categorized as active.

^b^Indicates a significant χ^2^ at the *P*<.001 level.

^c^Indicates a significant χ^2^ at the *P*<.05 level.

We then examined the differences in activity levels across the three app use groups using the IPAQ. Differences in each activity domain were explored using a between-groups analysis of variance (ANOVA). One-tailed planned contrasts were used to compare the current users to non-users and past users, reflecting our a priori hypothesized effect of greater activity levels for the current users. Means, standard deviations, *F*, and *P* values for all analyses are presented in Table 3. Overall differences between groups were found for total leisure METs, vigorous leisure METs, and walk leisure METs. Moderate leisure METs were marginally significant. No significant differences in activity levels were found in any other activity domain, including total METs, work METs, transportation to work METs, or home/gardening METs. Planned contrasts further showed that current users had higher activity levels compared to non-users in all leisure subdomains. Current users had higher activity levels compared to past users in walk leisure, and total vigorous leisure METs but not moderate leisure METs. The non-users and past users were not different from each other in any of the leisure activity subdomains.

**Table 3:** MET comparisons across app use groups.^a^

	Mean (SD)			*F* ^b^	*P* value	*P* value		
	Non-users	Past users	Current users			Current user vs non-user	Current user vs past user	Non-user vs past user
Total MET	3724 (4694)	2976 (4382)	4351 (5675)	2.1	.12	.04	.023	.19
Total Leisure MET	577 (1205)	612 (1518)	1169 (2088)	6.4	.002	.001	.009	.85
Vigorous Leisure MET	383 (896)	440 (1005)	730 (1439)	3.8	.02	.006	.034	.67
Walk Leisure MET	191 (395)	146 (521)	427 (729)	8	≤.001	≤.001	.001	.53
Moderate Leisure MET	77 (287)	105 (362)	162 (386)	2.6	.076	.024	.22	.49
Work MET	2976 (3435)	1796 (2378)	2564 (3160)	2.3	.1	.39	.22	.036
Transportation MET	564 (944)	524 (842)	633 (1130)	.277	.76	.56	.48	.77
House Work/ Gardening MET	1450 (2357)	1304 (2282)	1500 (2370)	.19	.83	.55	.85	.61

^a^Comparisons of current app users, those who began using an app and then stopped, and those who never used an app on the IPAQ self-reported metabolic equivalent of task, across eight activity categories. MET values reflect estimated totals for 7 days prior to study participation.

^b^Degrees of freedom are 2 and 512 for all reported omnibus *F* tests.

### Barriers to Exercise

In the next analysis, we examined how the use of exercise apps is associated with barriers to exercise. Percent of active participants, based on the exercise frequency measure in each of the three app groups, along with the omnibus chi-square, *P* values, and pairwise chi-square comparisons are presented in Table 2 separately for the high and low barriers groups. Low barrier participants were more likely to be active than high barrier participants across all three conditions. Both high and low barrier current app users were significantly more active than non-users. Critically, only the high barrier, but not the low barrier, current users were more active than past users. Past users and non-users were not different from each other independent of their high versus low barrier status. These results suggest that individuals with high barriers to exercise benefit more from using an exercise app, compared to individuals with low barriers to exercise.

One possible explanation for the finding that current app users exercise more than individuals who are not currently using an exercise app is that individuals who exercise more often may be more likely to buy exercise apps. Another possibility is that app use will be associated with more leisure time activity even among active participants who exercise on a regular basis. To examine this, in the next analysis we included only participants who indicated that they are active (ie, exercise at least 2 or 3 times a week). A 2 (high/low barriers) X 3 (non-users, past users and current users) independent-groups ANOVA was then conducted, with Total Leisure MET as the dependent variable. The results revealed a significant main effect of app usage (*F*_2,227_=3.1, *P*=.04). Additionally, the app usage X barriers interaction (*F*_2,227_=3.3, *P*=.04) remained significant. These results show that individuals with high barriers to exercise benefit more from using exercise apps, even among individuals who exercise regularly.

### Simple Moderation: Barriers as Moderators of Exercise

The analyses above suggest that there is an interaction between barriers and app use. To formally model this interaction, we carried out a moderation analysis with app use as a 3-level multi-categorical predictor (non-users, past users, and current users), barriers as the continuous moderator and exercise frequency as the outcome variable.

**Figure 2:**
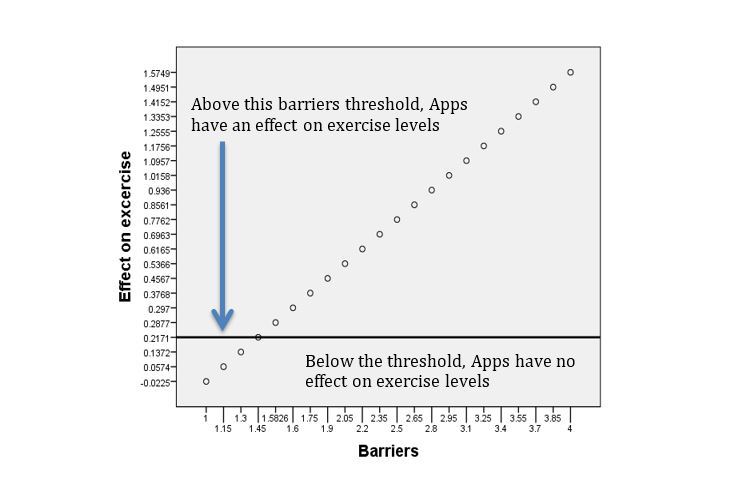
Relationship between app use and exercise at different levels of the continuous barriers to exercise moderator.

For all models below, all participants including those older than 35 were included in the analyses, with age as a covariate. The full model accounted for 29.7% of the variance in exercise frequency (*F*_6,700_=49.2, *P*<.001). The moderation analysis revealed that the association between app use and exercise levels was significantly moderated by barriers to exercise (R^2^ change due to the interaction=.0069, *F*_1,721_=6.8, *P*=.009). Specifically, app use was associated with higher levels of exercise for those participants who had more barriers. The Johnson-Neyman bootstrapping technique was used to probe the effect of app use at each level of the continuous moderator (see [34], pp. 234-56). As shown in Figure 2, the association between app use and exercise increases with higher barriers levels. The Johnson-Neyman bootstrapping technique further revealed that a barriers score of 1.45 was the cut-off point below which app use was no longer associated with increased exercise among current users compared to non-users.

The contrast between current users and non-users was the only one that resulted in significant moderation. The contrast between past users and non-users was not significant (R^2^ change due to the interaction=.0004, *F*_1,721_=.3637, *P*=.55). Most importantly, Johnson-Neyman bootstrapping illustrated that there were no significant changes in the association between app use and exercise levels at any level of the moderator.

### Simple Mediation: Exercise as a Mediator of Body Mass Index

**Figure 3:**
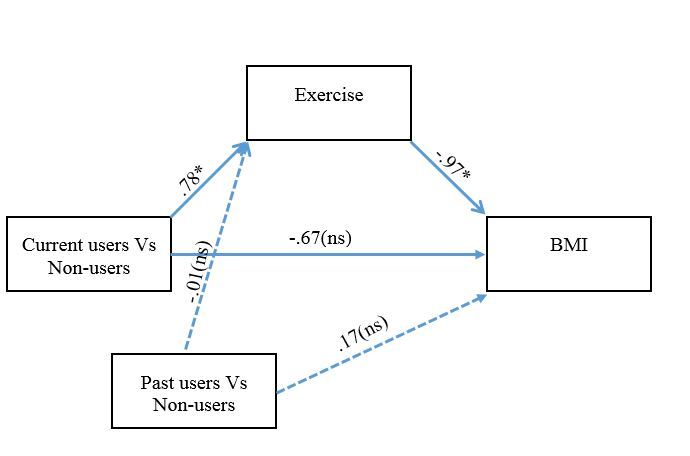
Mediation model in which app use was modeled as a 3-level predictor categorical variable in two separate contrasts. Solid lines depict the indirect and direct effects of a 2-level current users vs non-users contrast’s effect on BMI. The dashed lines depict the indirect and direct effects effect of a 2-level past users vs non-users contrast’s effect on BMI. Asterisk indicates significant results at *P*<.05 level. ns indicates non-significant results at the *P*<.05 level.

In the next model, we extended the hypothesized effect of app use from exercise to our measure of health status, represented by BMI. In this model (see Figure 3), exercise frequency was entered as a mediator between app use and BMI. The main goal of this mediation model was to test for (1) significant indirect effects of app use on BMI, through exercise levels and (2) to examine whether this indirect effect completely accounts for the association between app use and BMI (ie, no significant direct effect).

The weights for the paths in the model are presented in Figure 3. For the current users versus non-users contrast, the 95% confidence interval for the indirect effect ranged from -1.22 to -.43, indicating that the indirect effect was significantly different from zero at the *P*<.05 level. For the direct effect on the other hand, the 95% confidence interval ranged from -1.47 to 1.15, indicating that the direct effect was not significant. These results show that the reason that app users have lower BMI compared to non-users is that app users exercise more. The lack of a significant direct effect further shows that exercise fully accounts for the BMI differences between current users and those who never used exercise apps.

For the past users versus non-users contrast, the 95% confidence interval for the indirect effect ranged from -.25 to .29, indicating that the indirect effect was not significantly different from zero at the *P*<.05 level. The direct effect was likewise not significant, ranging from -1.14 to 1.48.

### Moderated Mediation: Barriers Moderate the Mediating Effect of Exercise on Body Mass Index

Next, we combined the moderation and mediation models to test a moderated mediation model, in which barriers to exercise moderate the indirect path from app use to BMI through exercise frequency (see Figure 1 above). The moderated mediation model built on the previous analyses showed that (1) app use is associated with decreased BMI, (2) app use is associated with increased exercise frequency, (3) the association between app use and exercise frequency is moderated by barriers to exercise, and (4) that the association of app use and BMI is mediated by exercise levels. The results confirmed the indirect effect of app use on BMI through increased exercise was moderated by barriers to exercise (95% CI -.0821 to -.006).

### Modeling Multiple Serial Mediation

**Figure 4:**
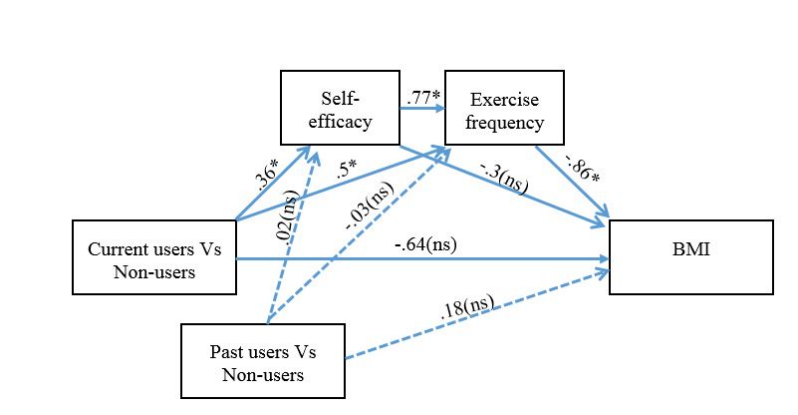
Mediated mediation model in which app use was modeled as a 3-level predictor categorical variable in two separate contrasts. Solid lines depict the indirect and direct effects of a 2-level current users vs non-users contrast’s effect on BMI. The dashed lines depict the indirect and direct effects effect of a 2-level past users vs non-users contrast’s effect on BMI. Asterisk indicates significant results at *P*<.05 level. ns indicates non-significant results at *P*<.05 level.

The previous analyses established that (1) app use is associated with lower BMI, (2) app use is associated with higher levels of exercise, and (3) that app use is associated with BMI through increased exercise levels. In the next model, we added self-efficacy as an additional plausible mechanism between app use and exercise levels, to examine whether the mediation effect of exercise is itself mediated by self-efficacy. Self-efficacy was added to the model as an additional mediator, which was serial and preceded the exercise frequency mediator in the causal chain (Figure 4). The main goal of this mediation model was to test (1) for serial mediation of the association between app use and BMI, through self-efficacy and exercise levels, (2) whether self-efficacy mediates the effect directly without exercise in the model, (3) whether this indirect serial mediation effect completely accounts for the association between app use and BMI and (4) whether self-efficacy precedes or follows exercise in the causal chain between app use and BMI.

For the indirect effect of exercise on BMI through self-efficacy and exercise frequency, the 95% confidence interval ranged from -.46 to -.1, indicating that the indirect effect for serial mediation was significantly different from zero at the *P*<.05 level. For the direct effect on the other hand, the 95% confidence interval ranged from -1.87 to .6, indicating that the direct effect was not significant. Reversing the order of the self-efficacy and exercise frequency mediators resulted in non-significant indirect effects for both contrasts.

These results show that app use is associated with increased self-efficacy, which in turn mediates exercise levels, accounting for lower BMI among current app users compared to non-users. Importantly the indirect effect of self-efficacy in the absence of exercise in the model was not significant. This shows that self-efficacy by itself is not a mediator of BMI, but that self-efficacy’s mediation of BMI occurs through its mediation of exercise.

When the past users group was entered as the independent variable with the current users groups entered as a covariate, both the direct and indirect effect were not significant (direct effect: -1.1 to 1.4; indirect effect -0.14 to 0.10). This indicates that past users are not different from non-users.

## Discussion

### Principal Findings

The goal of the current study was to examine whether individuals who use exercise apps exercise more than individuals who do not use such apps, and to identify plausible mechanisms by which the use of exercise apps may lead to increased levels of exercise and improved health outcomes. Current users of exercise apps were 27% more likely to self-report being active compared to participants who have either never used an exercise app or stopped using their apps. Increased activity among app users was specific to leisure time, including vigorous and moderate leisure activities, walking during leisure time, and total leisure time activities. Leisure time activity has been shown in exercise intervention studies to be the most important time for exercise. A meta-analysis of 127 studies showed that the most successful interventions are those that promote increased leisure time activity [35]. Leisure time activity, specifically, also has strong associations with health outcomes including reduced risk of dementia [36], metabolic syndrome [37], myocardial infarction [38], and death [39]. Importantly, no differences were found between current app users and non-users among other activity domains such as total MET or transportation to work. The lack of difference in overall activity shows that app users and non-users in our sample are not different in their overall activity profile, but differ specifically with regard to their leisure time exercise activities. These results show that mobile exercise app users use their leisure time in a more health-oriented way and suggest that mobile exercise apps may help users to increase their leisure time physical exercise activity levels.

Ideally, the three groups in this study represent a sequence of events in time. These results would provide insight as to what occurs when an individual transitions from not being an app user, to being an app user, and then to stopping their app use. Interpreted this way, these results indicate that using an app increases leisure exercise activity and leads to a reduction in BMI, and that stopping to use an app reverses these gains. However, the cross-sectional nature of this study precludes this interpretation. While our analyses utilized covariates such as age, income, and socioeconomic status, it is still possible that pre-existing differences such as interest in exercise can account for the differences between app adopters and non-adopters.

This interpretation is less likely to account for the differences observed between those app users who are currently using their app and those who are no longer using it. In particular, BMI among current users, but not among past users, was negatively correlated with how long they used their exercise app. This result is consistent with the interpretation that extended app use leads the user to exercise more consistently for extended time periods, which leads to reductions in BMI. However, it is still possible that higher interest in exercise, which may be more likely among individuals with lower BMI, can account for both the adoption of an app and the length of its use. It is thus still plausible that individuals who are more interested in exercise are more likely to seek out tools, such as exercise apps, which can help them to achieve their goals—but at the same time these individuals would be more likely to exercise even in the absence of such tools. In other words, app users may be natural “high exercisers”, who also have lower BMI.

To control for the potential bias in the disproportionally high number of high exercisers among current app users, we analyzed a subsample of participants who all reported to be active (ie, exercise at least two times per week). In this subsample of participants, app users still had higher levels of leisure time activity compared to non-users. These analyses reduce the likelihood that pre-existing attitudes toward exercise are able to account for the differences between current app users and non-users that were observed in this study. The models presented in this study put additional significant constraints on the plausibility of this alternative explanation because it was the app users with the highest number of barriers who were more likely to benefit from using an app both in terms of increased exercise levels and decreased BMI. High barrier individuals are generally likely to exercise least, and it is specifically among these individuals that the strongest effects of app use were observed. Although the sum total of our results suggest that there may be a causal relationship between app use and health outcomes, as in any cross-sectional study the possibility of confounding always exists. Future studies that use experimental and longitudinal study designs will be able to shed more light on the effects described in this study.

**Figure 5:**
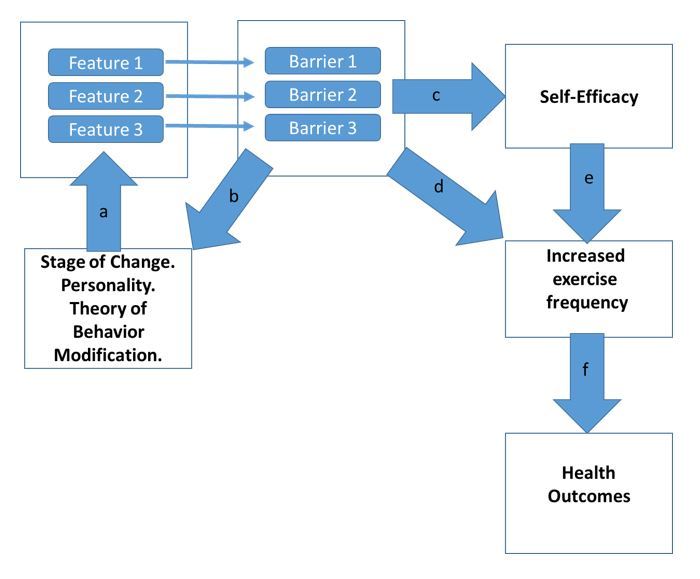
Barrier-centered model of exercise apps as exercise behavior-change intervention delivery systems. In this model, an individual’s barriers are taken into account during the app design process (b) personality, stages of change, and theory-driven approaches are all used in tailoring sets of features (a) that will maximally help the individual overcome their barriers. The effectiveness of the features leads to increased exercise frequency either directly (d) or through increased self-efficacy (c, e), leading to improved health outcomes (f).

Exercise interventions do not directly change behavior [40] but instead have their effect through intermediary variables referred to as mediators, and these effects are often bounded by moderators. To develop an understanding of the mechanisms that lead exercise app users to exercise more, and to explore the possible boundary conditions around these effects, we tested a number of mediation and moderation models. In the mediation models, the indirect effects provide insight as to the plausible mechanisms through which exercise apps, as interventions, may contribute to behavior change. We use the results of these models to develop a framework for understanding the effect of exercise apps as an exercise intervention of potentially high public health value and for contextualizing exercise apps within the framework of behavior change theories (see Figure 5).

### The Role of Barriers

While some people do not exercise due to a lack of interest and motivation, to a large degree lack of exercise is due to specific barriers that prevent people from exercising [41]. Barriers to exercise can be viewed as the common denominator and the overarching target that all exercise-focused interventions aim to address. For this reason, we chose barriers to exercise as the starting point in our models. At the broadest level, our models predicted that the effectiveness of apps comes from their ability to help individuals overcome their barriers.

Barriers were found to be significant moderators of app use on exercise. Individuals who have relatively few barriers were found to be less likely to benefit from exercise apps, likely because their engagement in exercise activity is not hampered by barriers, and these individuals are thus less in need of an intervention. Individuals with relatively high levels of barriers on the other hand were most likely to benefit from using exercise apps—an effect that extended to their BMI.

Common barriers to exercise include lack of time, lack of access to exercise facilities, lack of enjoyment of exercise, and/or lack of energy to exercise [42]. For example, an individual may be highly motivated to jog on a regular basis but may be prevented from doing so due to their traveling schedule [43]. Being in a novel location on a regular basis may make it difficult to find appropriate locations for jogging and exercising, thus making participation in consistent physical activity less likely over time. Frequent travel is an objective exercise barrier, and to the extent that an exercise app can provide tools with which to overcome that barrier, it may help the user to exercise more. For example, an app that incorporates a global positioning system (GPS) feature that tracks the user’s location and informs the user about nearby places to run or exercise may be able to help that individual overcome that particular barrier. Apps that provide information or video tutorials on proper exercise techniques can help overcome knowledge barriers, and these apps may be especially useful in certain special-needs populations such as pregnant women or individuals with disabilities [44], where special exercise techniques are often required for increased safety. Another example of how apps can help users overcome specific barriers is by features that help to motivate a user to increase their physical activity levels. For example, gamification apps may be able to improve motivation by increasing overall enjoyment of running [45]. Apps can also improve motivation by incorporating motivational messages, especially if those messages are based on tracking individual performance.

### Self-Efficacy

Exercise self-efficacy refers to a person’s confidence in their ability to engage in exercise [40]. Individuals who are more confident in their ability to exercise have been shown to be more physically active, and numerous studies have shown that belief in one’s ability to exercise is one of the best predictors of exercise performance. In a study of over 2500 people, self-efficacy was revealed to be the strongest predictor of vigorous exercise compared to 25 other predictors that were examined [46,47].

Self-efficacy both predicts and is predicted by exercise activity, and is likely to be in a causally reinforcing relationship with exercise [40]. At the cognitive level, interventions that are successful at increasing exercise levels also empower individuals to have confidence in their ability to follow an exercise regimen. This is consistent with two alternative views as to how the use of exercise apps may affect BMI through self-efficacy and exercise. One possibility is that app use increases self-efficacy, which increases exercise levels, leading to lower BMI. Another possibility is that app use increases exercise levels, which increase self-efficacy. Serial mediation allows for direct testing of these causal paths by changing the order of the serial mediators, because the predictor and outcome variable remain identical in the model and, thus, both models’ parameters can be compared directly [48].

Our results show that app use is associated with higher self-efficacy. Self-efficacy mediates the relationship between app use and exercise. Exercise in turn mediates the association between self-efficacy and BMI. Specifically, only the model in which self-efficacy was entered as the first mediator and exercise as the second mediator was significant, but not the model in which the exercise mediator preceded the self-efficacy mediator. Additionally, increased self-efficacy does not directly mediate reductions in BMI, but only through its mediation of increased exercise levels. These results show that self-efficacy is a central mechanism by which the use of exercise apps is associated with both exercise and health outcomes (BMI).

These results provide insight into the possible mechanisms through which apps, as interventions, may influence exercise and health. Self-efficacy interventions have been shown to causally improve exercise levels [49]. Additionally, self-efficacy is effective in helping individuals to overcome their barriers [50]. Increased levels of self-efficacy among app users may stem from the increased confidence the users obtain from using app features that help them overcome their barriers. To the extent that app use helps individuals to overcome their barriers, individuals’ self-confidence in their ability to consistently follow an exercise program will increase, and this increase in self-efficacy will be tied to increased exercise levels and positive health outcomes. In summary, our models show that individuals who use apps are more likely to exercise despite their barriers, which mediates increased self-efficacy and reduced BMI.

### Exercise Apps and Public Health

Over the last 50 years, the United States population has seen a significant reduction in overall physical activity [51]. Decreased demand on manual labor in the workforce and changes in transportation patterns have contributed to a significant reduction in non-leisure activity levels. While leisure time physical activity has remained largely stable and may even have slightly increased, the net activity levels in the population continue to decrease dramatically [51]. Interventions that can help to at least partially reverse this trend can have an immense impact on overall population-wide health outcomes, and health care expenditures. To that end, the development of effective interventions for increasing overall activity levels are a key aim of public health exercise science and policy.

Mobile apps have a number of advantages over other intervention delivery systems such as Internet websites [52] for promoting physical activity. One advantage of exercise apps is their degree of customizability. An app can incorporate features that are specific for the needs of an individual and, for example, can take individualized data such as connecting to a user’s calendar to help schedule exercise sessions. Apps are also mobile and able to provide services on a virtually continuous basis. Because people keep mobile phones with them most of the day, apps can also track activity, monitor progress, and deliver messages on an ongoing basis. The customizability, versatility, and portability of mobile apps make them ideal as potential intervention delivery systems.

The results presented in this study suggest that apps, as intervention delivery systems, have the potential to significantly improve population exercise levels and may thus have a significant impact on future public health outcomes. Within this framework, apps can be viewed as collections of features. Each feature targets specific barriers, and to the extent that a feature is successful at helping an individual overcome their barriers, it increases their self-efficacy with regard to that barrier and subsequently affects exercise behavior and health outcomes (see Figure 5).

While extant apps provide multiple features that appear to help users overcome their barriers, the effectiveness of exercise apps can be significantly improved by the utilization of individualized, theory-driven approaches (see Figure 5). There is wide recognition that the effectiveness of interventions is enhanced by the theories of behavior change [40]. Reviews of apps that are currently on the market however, reveal a profound lack of theory-driven and evidence-based approaches in app design. For example, in the Transtheoretical Model, barriers differ depending on the stages of change. Individuals in the preparation stage have more perceived barriers compared to those in the active stages. Further, in the active and maintenance stages, barriers that are associated with relapse are going to be of high importance. However, even the current top exercise apps currently do not have features aimed at relapse prevention [53], although such approaches are currently in development [19]. Additionally, apps should take personality differences into account, as personality traits such as conscientiousness, neuroticism, and extraversion are associated with exercise activity and with specific barriers [54,55]. For example, a low conscientiousness individual who is in the maintenance stage may be more likely to have a scheduling barrier. For this individual, features that address scheduling and incorporate reminders that utilize personalized calendar information, including holidays, appointments, and other commitments have the potential to deliver an individually tailored intervention that takes their needs into account. More research is needed in order to understand how best to incorporate theory into an individualized model of feature delivery. Overall, however, conceptualizing apps as interventions that deliver customizable, theory-driven, person-specific features for the purpose of overcoming barriers provides a helpful framework within which to approach the potential effectiveness of apps in future studies. More work needs to be done to ascertain how to best profile users on a regular basis in order to provide a more individualized feature delivery system and to maximize the match between features and their mode of presentation for each individual. This theory-driven individualized mode of feature presentation is likely to further improve the effectiveness of exercise apps.

## Appendix

Multimedia Appendix 1 Stimulus materials.

## Abbreviations

ANOVA: analysis of variance
BMI: body mass index
CI: confidence intervals
CU: current users
EBBS: Exercise Benefits/Barriers Scale
ECS: The Exercise Confidence Survey
EF: exercise frequency
GPS: global positioning system
IPAQ: International Physical Activity Questionnaire
MET: metabolic equivalent of task
MTurk: Amazon Mechanical Turk
NU: non-users
PA: physical activity
PU: past users
