# Supplementary material for: Mobile Exercise Apps and Increased Leisure Time Exercise Activity: A Moderated Mediation Analysis of the Role of Self-Efficacy and Barriers
Source: J Med Internet Res. 2015 Aug 14;17(8):e195. doi: 10.2196/jmir.4142 (PMC4642397; doi:10.2196/jmir.4142)
Supplement: Multimedia Appendix 1 [file jmir_v17i8e195_app1.pdf]

Stimulus materials

The following is a survey about exercise. Please answer the following questions as honestly as possible. Thank you for participating!!!

General Questions About Exercise

On average, how often do you currently exercise?

1. I don't currently exercise
2. I am exercising but infrequently (a few times a month at most)
3. I am exercising about once a week
4. I exercise 2 to 3 times a week
5. I exercise more than 3 times a week

What is the main reason you don't currently exercise?

6. I don't care about exercise
7. Exercise is not a priority for me
8. I don't feel like I need to exercise
9. I would like to exercise but have not gotten started
10. Other (please specify) ____________________

What is keeping you from getting started? Please check all that apply.

1. I don't know how or where to start
2. I don't have the time to exercise
3. I don't have the money I need to exercise
4. I don't have access to anywhere to exercise
5. I don't have the motivation to exercise
6. I am too tired to exercise
7. I am embarrassed to exercise
8. Exercise is not enjoyable
9. I have physical limitations which keep me from exercising
10. Other (please specify) ____________________

Why do you exercise? Choose all that apply.

11. To lose weight
12. To become more attractive
13. Overall health and longevity
14. Doctor's orders
15. It is enjoyable
16. I am training for a competition or an event
17. Other (please specify) ____________________

On average, how would you describe your exercise habits over the past 5 years?

11. No exercise
12. Infrequently (about 2 times a month)
13. Somewhat regularly (about once a week)
14. Regularly (2-3 times a week)
15. Frequently (more than 3 times a week)

General Questions about App Usage

Have you ever used an exercise application on any device (iPhone, iPad or Android)? An exercise application is a downloadable App for a mobile device. It is intended to help you exercise (note, apps that help with diet but not exercise are not considered exercise apps).

16. Yes
17. No

How many exercise apps have you used?

18. 0
19. 1
20. 2
21. 3
22. 4
23. 5
24. 6
25. 7
26. 8
27. more than 8

Below is a list of exercise apps. Please select the apps which you have used.

18. 7 Minute Workout “Seve...
19. 100 push ups challenge
20. 100+ Fitness Exercises

21. 100+ Fitness Exercises Free
22. 101 Yoga Poses
23. 10K Runner: 0 to 5K to 10K run..
24. 10K Trainer FREE - Run for PINK…
25. 10k Trainer Pro- Run for Pink
26. 200 Exercises- Bodyweight...
27. 200 Situps Challenge
28. 30 Day Squat Challenge Free
29. 300 Kettlebell Challenge: The…
30. 365days Push-ups
31. 5 in 1 Journal: Food, Sleep,…
32. 5 Minute Home Workouts-...
33. 500 Bodyweight Challenge: The…
34. 5K Runner: 0 to 5K run training
35. 5k Runner: 0 to 5k run training,.
36. 6 Pack Promise Plus
37. 6 Pack Promise(no s)
38. 7 Min Workout
39. 7 minute workout
40. 7 Minute Workout "Seve..
41. 7 Minute Workout (High..
42. 7-minute Scientific…
43. 8 Minute Workout
44. 90 Day Workout Tracker 1

45. 90 Day Workout Tracker 2
46. Ab Fitness exercises- Los...
47. Ab Trainer : 100+ ab exercises and
48. Ab trainer X free - six p...
49. Ab trainer x free hd - six...
50. Ab workout free - abdominal crunch...
51. Ab Workout Pro- Abdominal
52. Ab Workout Trainer - Steel…
53. Ab Workout X FREE - SixPack
54. Ab Workouts Express
55. Ab workouts free
56. Ab Workouts MMA FREE-Ji...
57. Ab Workouts Pro
58. Abs trainer free - exercise for pink
59. Abs Trainer pro- Exercise For PINK
60. Abs video workout app -...
61. Abs workout
62. Abs Workouts Getting A Perfect...
63. Absolute Abs
64. Academy Sports + Outdoors (VISA or LiveFit) This is for LiveFit
65. ActiveTravel
66. Adrian James 6 Pack Abs…
67. Air Pedometer
68. All-in Fitness 1000 Exercises...

69. All-in Tacker: Running, Cycli…
70. AllSport GPS LE
71. Alpha Trainer: Get Customize...
72. Arm Exercises- Personal Train...
73. Arm trainer: 100+ arm...
74. Arm Workout x Free- Bicep...
75. Arm Workouts - Owning Perfect…
76. Arm Workouts - Sculpting Perfe…
77. Arm workouts free
78. Authentic Yoga with Deepak…
79. AXA Health Gateway
80. Back Trainer 100+ back...
81. Ball & Band Lite by Powworkout
82. Ballet body fit by prima pilates
83. Ballstrike
84. Beat Training
85. BeatBurn Treadmill/Outd…
86. Belly Fat Workout Free
87. Beyond the Whiteboard
88. Bit of Exercise Lite- Running...
89. Blogilates official app
90. BodBot
91. Body Fitness - ultimate exercise
92. Body Fitness FREE -

93. Bodybuilding.com
94. Bodybuilding: Ab Workouts Plan
95. Bodybuilding: Back Exercises
96. BodyFate Workout
97. BodyMedia Fit
98. BodySpace
99. Bodyweight Training: you A...
100. BodyWeight workout Free
101. Brazilian Butt - best butt lift workout
102. Butt Exercises- Personal Train-
103. Butt lift workout hd
104. Butt Trainer: 100+butt...
105. Butt Workout
106. Butt workout free
107. Butts trainer free exercise for pink
108. Butts Trainer Pro - Exercise for…
109. C25K - 5K Trainer FREE
110. Calisthenics Kingz
111. Calistix FREE Personal Train…
112. Cardio - Heart Rate Monitor, 7…
113. Cardio Fitness Exercises-...
114. Cardio Workouts Free
115. Ches fitness exercises...
116. Chest Trainer: 100+chest...

117. Chest Workouts
118. Chest Workouts Free
119. Complete Gym Guide Lite
120. Core Workouts Free
121. Core Yoga
122. COREwheels by COREwheel…
123. Couch to 5k
124. Couch to 5k workout
125. Cross Trainer X FREE - Aerobic…
126. Cybex Bravo Workout Guide
127. Cycle Tracker Pro -…
128. Cyclemeter GPS - Cycling…
129. Daily Ab Workout
130. Daily Ab workout free
131. Daily Arm Workout
132. Daily arm workout free
133. Daily burn - video workouts
134. Daily Butt Workout
135. Daily Butt workout free
136. Daily Carb Premium-...
137. Daily cardio work out free
138. Daily Cardio Workout
139. Daily Leg Workout
140. Daily leg workout free

141. Daily Workouts
142. Daily Workouts FREE
143. Daily Yoga
144. Daily Yoga™
145. Dance Fitness Lite
146. Deck of Cards Workout
147. DesignYourBody
148. Diabetes In Check
149. Diet & Exercise Assistant
150. Digifit iCardio Multi-Sport He…
151. Distance Meter Free
152. Ease into 5k- Free, run walk...
153. Easy Gym Log
154. Easy Weights Log
155. Eatright 90- Nutrition log...
156. Endomondo Sports Tracker..
157. Enjoy exercise with hypnosis
158. Epic Workout Planner
159. Everybody Walk!
160. EveryMove Rewards For...
161. Everywhere exercise - evex
162. Everywhere exercise - evex stealth
163. Everywhere exercise - evex...
164. Everywhere Exercise-EvEx...

165. Exercise Calculator
166. Exercise calorie calculatior
167. Exercise- Interval Timer
168. Exercise Map Lite
169. Exercise reminder hd lite
170. Exercise tracker
171. Exercise.com
172. Extreme bootcamp! Wit...
173. Finess Buddy for iPad : 1700+…
174. Fit Friendzy - Exercise…
175. Fitbit
176. FitClick Diet & Workout Tracker
177. FitDeck+ BodyWeight
178. Fitfrnd - the best weight...
179. FitGeanie
180. fitID discover, track & share…
181. Fitlist - Free Fitness Tracker…
182. FitMagic-Exercise counter
183. Fitness Buddy FREE: 1700+ Exercise Workout Journal
184. Fitness Buddy FREE: 300+ Exercise Workout Journal
185. Fitness Builder
186. Fitness excercises in the office HD
187. Fitness Fast - daily fitness exercise...
188. Fitness HD: 700 exercise, Yoga...

189. Fitness Muscle
190. Fitness point - workout & Ex...
191. Fitness Point Pro - Workout &…
192. Fitness Pro
193. Fitness: Chest Workout Plans
194. Fitnessclass
195. FitnessKids
196. Fitnorama Magazine.
197. Fitstar: tony gonzalez
198. Flat Stomach & Abdomen…
199. Fleetly Fitness
200. Footsteps - pedometer free
201. Full Fitness : Exercise Work
202. Full fitness hd for ipad
203. Full fitness: exercise workout trainer
204. Gain fitness cross trainer-...
205. Game fit bike race - exercise power...
206. GameFit Bike Race PRO-
207. GameFit Racing (Exercise...
208. Get Active!
209. Get In Gear
210. Get Motivated to Exercise with...
211. Get Running (couch to 5k)
212. Get your body back after baby birth

213. GetHealth
214. Gorilla Workout : Fitness Aerobic,,
215. Gorilla Workout Free : Athletic..
216. Guy's Workout..
217. Gym Ball Workouts
218. Gym free: bodyweight...
219. Gym geek workout and...
220. Gym genius Workout Track-...
221. Gym hero - workout log & exercise..
222. Gym training by weightplan.com
223. Gym Training Lite by...
224. GymGoal 1 (old)
225. GymGoal Free
226. Gympact - cash reward to...
227. GymPad Exercise Fitness &...
228. HCTV
229. High Intensit Interval Trainer
230. hip and Thigh Workouts Free
231. Home Fitness Free
232. Hot Body Fitness with Amanda...
233. Hot5 Fitness : Video Workout…
234. How Do I Lose Weight Now-...
235. Human: Move 30 minutes or more
236. Hundred PushUps

237. I Want Six Pack Abs Version 2.0
238. iBelly Workout
239. Ibiker | Indoor and Outdoor...
240. Ideserve2
241. iExercise Journal FREE
242. iHealth MyVitals
243. iMuscle 2
244. IMuscle 2- iphone Edition
245. In Shape: Your Personal Coac...
246. Instant Fitness : 600+ exercises..
247. Intense 7 Mins Exercise
248. Interval Timer - Seconds by…
249. Interval timer - timing for hiit training...
250. Interval timer pro lite
251. Interval Timer Workout &…
252. iPedometer Lite
253. iPersonalTrainer - Fitness…
254. iPersonalTrainer HD- Fitness...
255. ipushups - the ultimate fitness...
256. iRehab Back Pain
257. iRunner l running...
258. iStepLog
259. iTooch 6th Grade Health…
260. iTreadmill:Pedome Ultra w/...

261. Iworkout Lite
262. iYoga Premium
263. iYoga+ hd
264. iYogaGuru Lite
265. JEFIT PRO Workout-...
266. JEFIT Workout - Free Personal Trainer...
267. Jillian michael's slim down solution
268. JIM Stoppani
269. Jog.fm - workout music at your...
270. JUMP ROPE WORKOUT
271. Jump Start - simple workout
272. Kettle Bell Workout 360…
273. Ladies' Ab Workout FREE
274. Ladies' Ab Workout HD
275. Ladies' Arm Workout FREE
276. Ladies' Back Workout FREE
277. Ladies' Butt Workout FREE
278. Ladies Butt Workout HD
279. Ladies' Leg Workout FREE
280. Ladies' Waist Workouts FREE
281. Ladies Workout Express
282. Learn Pilates
283. Learn Pilates Free
284. Leg Exercises - Personal Train…

285. Leg trainer: 100+ leg...
286. Leg Workouts - Striking A Perf…
287. Leg Workouts Free
288. Legs trainer free - exercise for pink
289. Legs Trainer Pro - Exercise for…
290. Legs Workout Lite
291. LIVESTRONG.COM - Calorie Track..
292. LiveWell Richmond
293. LogYourRun - GPS pedomete…
294. Lose the belly (weight loss fo... 2
295. Map My Fitness - Workout Trainer
296. Map My Fitness+ - Workout Trainer
297. Map my Ride- GPS Cycling
298. Map my Ride+- GPS Cycling
299. MapMyHike GPSHiking
300. MealLogger: Photo Food…
301. Mens Exercise...
302. Men's fitness complete spor...
303. Metricme - Workout, fitness, and Exercise...
304. miCoach
305. MiFitLife
306. MoPix Mobile- Watch movies
307. Motion Traxx Radio: Workout
308. Muscle & Fitness Trainer

309. Muscle Mag's 63 Best Ab...
310. Music Workout- Interval Timer f...
311. My Diet Coach - Weight Loss for..
312. My Diet Diary-Calorie Counter
313. My Pedometer: Ulti…
314. Myfit fitness - workout logger...
315. myWod- All-in-one Wod Logf...
316. Navy Seal Fitness
317. Nexercise - motivation to lose weight, to...
318. Nice Weight Loss Program-Your..
319. Nike training club
320. Nike+ fuelband
321. Nike+ Running
322. Nike+ Training
323. Nutrisystem
324. Nutrition Menu - Calorie, Exerci…
325. Office- Fit
326. Out-A-Bout
327. OZOM Track
328. Pacer - Pedometer plu..
329. Pcket Yoga
330. Pedometer
331. Pedometer Free
332. Pedometer HD Lite

333. Pedometer Lite
334. Pedometer++
335. Perfect Pilates Body with Andr..
336. Personal Workout
337. Physique workout tracker
338. PhysiqueBuild
339. Pilates
340. Pilates Anytime
341. Pilates Core by Method Yoga-..
342. Pilates Fitness Exercises - Cor…
343. Pilates Interactive
344. Pilates Workouts
345. Pilates!
346. Pocket yoga
347. Pocket Yoga- Practice builder
348. Points calculator & weekly weig...
349. Points calculator & weekly weight l...
350. Points Calculator for Wight Los...
351. Polar Beat - Sports & Fitness…
352. Pregnancy Exercise-..
353. Pregnancy Workout Advisor
354. Primal Paleo: gluten free, wod…
355. Prospex Athletic Training System
356. PT Timer Lite: Stretch &...

357. PT Timer: Stretch & Exercise
358. PumpNlog - Workput Log F…
359. Pumpup - fitness coach
360. Push ups free
361. Push Ups Trainer FREE
362. Push-Up Counter (free)
363. Pushup Fitness Exercises-...
364. Pushups 0 to 100 Exercise Work..
365. Pushups 0 to 100 exercise workout..
366. Pushups 100+ Lite
367. Pushups coach Free- Do 100...
368. Pushups trainer FREE - exercise for pink
369. PushUps Trainer Pro- Exercise f...
370. RANDOM WOD FREE
371. Random WOD Workout..
372. Ranger School Fitness - Army..
373. Rehabilitation For Lower Limbs
374. Remix Workouts
375. Road Warrior Workout by Nat…
376. Rob Riches Pro Workout Lite
377. Run 10k - interval training coach …
378. Run 5K - interval training coach…
379. Run Tracker Pro - TrainingPeak
380. Run with Map My Run- GPS…

381. Run with Map My Run+- GPS…
382. RunKeeper- GPS Track Running
383. Runmeter GPS - Running Joggi…
384. Running for Weight Loss…
385. Running Log Free
386. Runtastic gps running, walking
387. Runtastic Pedometer Ste..
388. Runtastic Pedometer Ste...
389. Runtastic PRO GPS Running
390. Runtastic Pull Ups Trainer
391. Runtastic Pull Ups Trainer Pro
392. Runtastic Push Ups Trainer
393. Runtastic Pushups Trainer Pro
394. Runtastic road bike gps cycli...
395. RuNtastic Sit Ups Trainer
396. Runtastic Sit Ups Trainer PRO
397. Runtastic Six Pack: Abs..
398. Runtastic Squats trainer
399. Runtastic Squats Trainer PRO
400. Sculpt My Pregnancy
401. See Me Get Fit - Record Change..
402. SELF Workouts
403. Shake Shake Pop Lite
404. Short sequence kids' yoga

405. Shoulder Fitness Exercises-...
406. Shoulder Trainer: 100+ shoulder...
407. Simple Tabata
408. Simply Yoga
409. Simply Yoga FREE
410. Situps 0 to 200: Abs Workout..
411. Situps 0 to 200: Abs Workout..
412. Situps Coach Free- Do 200 Situps...
413. Six Pack Abs- Personal Trainer
414. Six Pack Abs Workout FREE..
415. Sixpack - Personal Trainer
416. SixPack App PRO- Fitness...
417. Slim Body Fitness with…
418. Slim Plate: Weight Loss Portion...
419. Spin It - Random Exercise &…
420. Spinning | Indoor Cycling …
421. Sports Tracker
422. Spotter by Golds Gym
423. Squats Coach Free- Do 200...
424. Star Trainer Premium
425. StepTrakLite
426. Strava Cycling - GPS Biking and..
427. Strava Run- GPS Running, Training
428. Strong Runner- Running...

429. Sworkit - Circuit Training..
430. Sworkit Pro
431. Taabata Stopwatch Pro...
432. Tabata exercise interval timer free
433. Tabata Pro - Tabata Timer
434. Tabata Timer
435. Tabata-Timer
436. TactioHealth (Weight Loss,…
437. Tap & Track Calorie Counte...
438. TC 3-22.20- Army Physical...
439. Teemo: the fitness adventure...
440. The 7 minute workout
441. The FPR Prenatal Core…
442. The perfect muscle exercis…
443. The spartan 300 workout
444. The Wherever Workout
445. THI Personal Trainer Lite
446. Thirtyaday fitness
447. TK move body fitness - video...
448. Total Body Workout @
449. Touchfit: GSP 500+ Exercise …
450. Tracker for 25 Minute Workout
451. Tracker2GO Calorie Countin...
452. Tracknburn calorie, diet and exercise...

453. TracknShare- A universal life...
454. Tracknshare lite - a self he...
455. TrailMix Pro- Walk or run to...
456. Trails Lite GPS Tracker
457. Trainerize
458. Training Peaks
459. Trim & Tone Lite by Powerkout
460. TRX FORCE
461. Two Hundered Squats
462. Ultimate Core Workout - Free…
463. Ultimate Workout 2 Free - The Ne..
464. Ultimate Workout 3 Free Body..
465. Ultimate workout free - daily...
466. Unlimited Abs
467. UP by Jawbone
468. Virtual trainer
469. Virtual Training
470. WakeMyMojo
471. Walk n' Play
472. Walk Tracker Pro
473. Walk with Map My Walk- GPS..
474. Walk with Map My Walk+- GPS..
475. Walkmeter GPS - Walking Hiking..
476. Weekly Sit Ups Workout

477. Weight Lifting Videos: Learn...
478. Weight Loss
479. Weight Watchers Mobile
480. Weighttraining.com - free workout...
481. Winsor Pilates
482. Withings Health Mate
483. WOD - Workout Logger
484. Womens Health Lite
485. Work-Out
486. Workout (7 Minute Body…
487. Workout and Fitness Exercisees
488. Workout Coach- Manages Your
489. Workout Daily- Personal Fitness
490. Workout Hero - WOD and Fitness..
491. Workout log - fitness and exercise tracker...
492. Workout Log! - Exercise Tracker
493. Workout Mate Free
494. Workout of the day daily fitness exercise
495. Workout Plan - Fitness Schedule
496. Workout Trainer
497. Workout: Coach Fitness Video
498. WorkoutJournal for iPad
499. Yoga & Pilates with Kristin...
500. Yoga 101
501. Yoga at Home Videos for...
502. Yoga Fitness 3D
503. Yoga Fitness Poses -…
504. Yoga for Weight Loss
505. Yoga Library for iPad
506. Yoga STRETCH Lite
507. Yoga Studio
508. Yoga Trainer Lite
509. Yoga with Tania Lite
510. Yogify
511. Zombies, Run!
512. Zumba Dance
513. None of the above

If the app/s that you used were not on the above list, please list them here.

We will now ask you some questions about the app you used. If you have used more than 1, please make your responses based on the last app that you used. In the field below please tell us which app this was.

When did you start using the app? (Please select month and year)

Month	28. J	29. F	30. M	31. A	32. M	33. J	34. J	35. A	36. S	37. O	38. N	39. D
Year	40. 2	41. 2	42. 2	43. 2	44. 2	45. 2	46. 2	47. 2	48. 2	49. 2	50. 2	51. 2

Are you still using the app? Not using the app means that you either have no plans of using it in the future, or that you have not used it in the last month.

52. Yes
53. No

When did you stop using the app?

Month	54. J	55. F	56. M	57. A	58. M	59. J	60. J	61. A	62. S	63. O	64. N	65. D
Year	66. 2	67. 2	68. 2	69. 2	70. 2	71. 2	72. 2	73. 2	74. 2	75. 2	76. 2	77. 2

How long did you use the app?

78. 1 week or less
79. 2 weeks
80. 3 weeks
81. 4 weeks
82. 5 weeks
83. 6 weeks
84. 7 weeks
85. 8 weeks
86. 9 weeks
87. 10 weeks
88. 11 weeks
89. 12 weeks
90. 3 months
91. 4 months
92. 5 months
93. 6 months
94. 7 months
95. 8 months
96. 9 months
97. 10 months
98. 11 months
99. 1 year
100. 1 year, 1 month
101. 1 year, 2 months
102. 1 year, 3 months
103. 1 year, 4 months
104. 1 year, 5 months
105. 1 year, 6 months
106. 1 year, 7 months
107. 1 year, 8 months
108. 1 year, 9 months
109. 1 year, 10 months
110. 1 year, 11 months
111. 2 years
112. More than 2 years

How consistently did you use the app over this period of time?

113. Consistently (pretty much every time I exercise)
114. Inconsistently (I use it on and off when I exercise)
115. Very consistently in the beginning but then slowed down
116. Barely used it at all (I tried it a few times)

Why did you start using the app? Please choose all that apply.

514. To help me get started working out
515. To help me be consistent on maintaining my fitness
516. To help me intensify my workout
517. To help me focus more on a specific area (e.g. abs)
518. To learn an exercise routine or specific exercises
519. To monitor my activity and progress
520. The technology was "cool"
521. Someone recommended it
522. For its social aspects (e.g., facebook, twitter)
523. For scheduling and planning my workouts
524. Because it had a quick workout (e.g. 7 minutes)
525. To help find facilities or locations to exercise
526. For motivational purposes
527. To make exercise more enjoyable
528. To find exercises specifically for my health status (e.g. pregnancy, diabetes etc...)
529. Other ____________________

Why did you stop using the app? Please choose all that apply.

530. I got bored
531. I did not think the app was accurately recording my progress
532. I did not think the app was helpful
533. The app wasn't user friendly
534. The app had served its purpose
535. I stopped exercising
536. I lost access to the app (e.g. I lost my phone)
537. I am still using the app
538. Other ____________________

Did the app only focus on physical activity or did it address other issues as well (e.g., dieting, mental health, meditation, spirituality)?

117. Physical Activity only
118. Physical Activity Plus

Did you use the additional features (e.g., dieting, mental health, meditation, spirituality) of the app?

119. Yes
120. No

How often did you use the additional features (e.g., dieting, mental health, meditation, spirituality) of your app?

121. 10% of the time
122. 25% of the time
123. 50% of the time
124. 75% of the time
125. 100% of the time
126. None of the time

Did you feel that using the additional features (e.g., dieting, mental health, meditation, spirituality) of the app helped you to become more physically active?

127. Yes
128. No

What population is the app intended for?

129. General Population (everyone)
130. Specific Population (such as the elderly, children, women etc..)
131. Clinical Population (such as people with diabetes, the obese, pregnancy etc...)

What is the specificity of the exercises that the app addresses?

132. Highly Specific - Only helped with one exercise such as push ups, sit ups etc...
133. Specific - Multiple exercises to address 1 issue or area of the body such as chest, arms, legs etc...
134. General - Overall Health such as exercises that address multiple areas, running etc...

What type of physical activity did your app focus on?

135. Aerobic exercise (e.g., weight loss, cardiovascular conditioning)
136. Anaerobic exercise (e.g., weight training, high intensity interval work, speed, power)
137. A combination of aerobic and anaerobic exercise

Does/did the app utilize an external device that your wore on your wrist or hip to provide feedback (e.g, Nike Fuelband, Jawbone Up, Fitbit)?

138. Yes
139. No

Do you feel that the use of the external device (e.g, Nike Fuelband, Jawbone Up, Fitbit) enhanced the use of the app?

140. Definitely yes
141. Probably yes
142. Probably not
143. Definitely not

Do you feel that the use of the external device (e.g, Nike Fuelband, Jawbone Up, Fitbit) increased your level of physical activity?

144. Definitely yes
145. Probably yes
146. Probably not
147. Definitely not

Does/did the app have a structured exercise program with progressive goals that build off each other?

148. No
149. Yes - Goals set by app
150. Yes - Goals set by user

Does/did the app have any social aspects (e.g. links to Facebook or connects you with other app users)?

151. No
152. Yes - through an outside social media website (e.g. Facebook or Twitter)
153. Yes - through the app itself
154. Yes - through both the app and an outside website

Does/did the app provide feedback (i.e., show your progress or contact you to let you know how you are doing) based on your performance?

155. Yes
156. No

Did the app provide feedback that was "proactive" such as a message like "Good Job" would appear on your screen, or did you just have the option of checking your feedback (such as your progress)?

157. The app provided proactive messages when my information was uploaded.
158. The app did not provide proactive messages but I had an option to check my feedback.

The feedback offered by the app helped me to exercise more.

159. Strongly Disagree
160. Disagree
161. Agree
162. Strongly Agree

In addition to feedback on your day's activity, did the app provide any reminders like "don't forget you have a 1PM workout session tomorrow"?

163. The app provided reminders of upcoming workout sessions.
164. The app did not provide reminders.

Did the app provide you with a method of inputting data?

165. No
166. Yes - Manually (you enter data, such as reps, time etc..., manually)
167. Yes - Automatically (i.e., the app automatically tracks data)
168. Yes - both Manually and Automatically

How certain are you that your app is accurate in recording how much exercise you do (steps taken, heart beat etc.)?

169. I am fairly certain that my app is inaccurate when recording how much exercise I do.
170. I have at times doubted the accuracy with which my app records how much exercise I do.
171. I have never doubted whether my app is accurate when recording how much exercise I do.
172. I am fairly certain that my app is always accurate when recording how much exercise I do.

Please tell us if the app had any other features that you felt were important. (Please note that we give bonuses for meaningful responses).

Barriers/Benefits Scale

Below are statements that relate to ideas about exercise. Please indicate the degree to which you agree or disagree with each statement.

I enjoy exercise.

173. Strongly Disagree
174. Disagree
175. Agree
176. Strongly Agree

Exercise decreases feelings of stress and tension for me.

177. Strongly Disagree
178. Disagree
179. Agree
180. Strongly Agree

Exercise improves my mental health.

181. Strongly Disagree
182. Disagree
183. Agree
184. Strongly Agree

Exercising takes too much of my time.

185. Strongly Disagree
186. Disagree
187. Agree
188. Strongly Agree

I will prevent heart attacks by exercising.

189. Strongly Disagree
190. Disagree
191. Agree
192. Strongly Agree

Exercise tires me.

193. Strongly Disagree
194. Disagree
195. Agree
196. Strongly Agree

Exercise increases my muscle strength.

197. Strongly Disagree
198. Disagree
199. Agree
200. Strongly Agree

Exercise gives me a sense of personal accomplishment.

201. Strongly Disagree
202. Disagree
203. Agree
204. Strongly Agree

Places for me to exercise are too far away.

205. Strongly Disagree
206. Disagree
207. Agree
208. Strongly Agree

Exercising makes me feel relaxed.

209. Strongly Disagree
210. Disagree
211. Agree
212. Strongly Agree

Exercising lets me have contact with friends and persons I enjoy.

213. Strongly Disagree
214. Disagree
215. Agree
216. Strongly Agree

I am too embarrassed to exercise.

217. Strongly Disagree
218. Disagree
219. Agree
220. Strongly Agree

Exercising will keep me from having high blood pressure.

221. Strongly Disagree
222. Disagree
223. Agree
224. Strongly Agree

It costs too much to exercise.

225. Strongly Disagree
226. Disagree
227. Agree
228. Strongly Agree

Exercising increases my level of physical fitness.

229. Strongly Disagree
230. Disagree
231. Agree
232. Strongly Agree

Exercise facilities do not have convenient schedules for me.

233. Strongly Disagree
234. Disagree
235. Agree
236. Strongly Agree

My muscle tone is improved with exercise.

237. Strongly Agree
238. Agree
239. Disagree
240. Strongly disagree

Exercising improves functioning of my cardiovascular system.

241. Strongly Disagree
242. Disagree
243. Agree
244. Strongly Agree

I am fatigued by exercise.

245. Strongly Disagree
246. Disagree
247. Agree
248. Strongly Agree

I have improved feelings of wellbeing from exercise.

249. Strongly Disagree
250. Disagree
251. Agree
252. Strongly Agree

My spouse (or significant other) does not encourage exercising.

253. Strongly Disagree
254. Disagree
255. Agree
256. Strongly Agree
257. Not applicable

Exercise increases my stamina.

258. Strongly Disagree
259. Disagree
260. Agree
261. Strongly Agree

Exercise improves my flexibility.

262. Strongly Disagree
263. Disagree
264. Agree
265. Strongly Agree

Exercise takes too much time from family relationships.

266. Strongly Disagree
267. Disagree
268. Agree
269. Strongly Agree

My disposition is improved with exercise.

270. Strongly Disagree
271. Disagree
272. Agree
273. Strongly Agree

Exercising helps me sleep better at night.

274. Strongly Disagree
275. Disagree
276. Agree
277. Strongly Agree

I will live longer if I exercise.

278. Strongly Disagree
279. Disagree
280. Agree
281. Strongly Agree

I think people in exercise clothes look funny.

282. Strongly Disagree
283. Disagree
284. Agree
285. Strongly Agree

Exercise helps me decrease fatigue.

286. Strongly Disagree
287. Disagree
288. Agree
289. Strongly Agree

Exercising is a good way for me to meet new people.

290. Strongly Disagree
291. Disagree
292. Agree
293. Strongly Agree

My physical endurance is improved by exercising.

294. Strongly Disagree
295. Disagree
296. Agree
297. Strongly Agree

Exercising improves my self-confidence.

298. Strongly Disagree
299. Disagree
300. Agree
301. Strongly Agree

My family members do not encourage me to exercise.

302. Strongly Disagree
303. Disagree
304. Agree
305. Strongly Agree

Exercising increases my mental alertness.

306. Strongly Disagree
307. Disagree
308. Agree
309. Strongly Agree

Exercise allows me to carry out normal activities without becoming tired.

310. Strongly Disagree
311. Disagree
312. Agree
313. Strongly Agree

Exercise improves the quality of my work.

314. Strongly Disagree
315. Disagree
316. Agree
317. Strongly Agree

Exercise takes too much time from my family responsibilities.

318. Strongly Disagree
319. Disagree
320. Agree
321. Strongly Agree

Exercise is good entertainment for me.

322. Strongly Disagree
323. Disagree
324. Agree
325. Strongly Agree

Exercising increases my acceptance by others.
326. Strongly Disagree
327. Disagree
328. Agree
329. Strongly Agree

Exercise is hard work for me.
330. Strongly Disagree
331. Disagree
332. Agree
333. Strongly Agree

Exercise improves overall body functioning for me.
334. Strongly Disagree
335. Disagree
336. Agree
337. Strongly Agree

There are too few places for me to exercise.
338. Strongly Disagree
339. Disagree
340. Agree
341. Strongly Agree

Exercise improves the way my body looks.
342. Strongly Disagree
343. Disagree
344. Agree
345. Strongly Agree

<div>Exercise has little value for my health and fitness.</div>
346. Strongly Disagree
347. Disagree
348. Agree
349. Strongly Agree

Health outcomes are out of my control.
350. Strongly Disagree
351. Disagree
352. Agree
353. Strongly Agree

I don’t know how to exercise or operate the necessary equipment.

354. Strongly Disagree
355. Disagree
356. Agree
357. Strongly Agree

I am scared that I will get injured while exercising.

358. Strongly Disagree
359. Disagree
360. Agree
361. Strongly Agree

I lack self-management skills, such as the ability to set personal goals, monitor my progress, or reward my progress toward such goals.

362. Strongly Disagree
363. Disagree
364. Agree
365. Strongly Agree

Questions about how Apps Help to Overcome Specific Barriers

Below are statements about your apps influence on your exercise. Please indicate the degree to which you agree or disagree with each statement.

My app helped me to get up early, even on weekends, to exercise.

366. Strongly Disagree
367. Disagree
368. Neither Agree nor Disagree
369. Agree
370. Strongly Agree
371. Not Applicable

My app helped me see how exercise has value to my health.

372. Strongly Disagree
373. Disagree
374. Neither Agree nor Disagree
375. Agree
376. Strongly Agree

My app helped me to stick to my exercise program after a long, tiring day at work.

377. Strongly Disagree
378. Disagree
379. Neither Agree nor Disagree
380. Agree
381. Strongly Agree
382. Not Applicable

My app helped me to exercise even though I was feeling depressed.

383. Strongly Disagree
384. Disagree
385. Neither Agree nor Disagree
386. Agree
387. Strongly Agree
388. Not Applicable

My app helped to set aside time for a physical activity program (e.g., walking, jogging. swimming, biking, or other continuous activities) for at least 30 minutes, 3 times per week.

389. Strongly Disagree
390. Disagree
391. Neither Agree nor Disagree
392. Agree
393. Strongly Agree
394. Not applicable

My app helped me to stick to my exercise program when undergoing a stressful life change (e.g., divorce, death in the family, moving).

395. Strongly Disagree
396. Disagree
397. Neither Agree nor Disagree
398. Agree
399. Strongly Agree
400. Not Applicable

My app helped me exercise first and play later (e.g., attend a party only after exercising).

401. Strongly Disagree
402. Disagree
403. Neither Agree nor Disagree
404. Agree
405. Strongly Agree

My app helped me to stick to my exercise program even when I had excessive demands at work.

406. Strongly Disagree
407. Disagree
408. Neither Agree nor Disagree
409. Agree
410. Strongly Agree
411. Not Applicable

My app helped me to stick to my exercise program when social obligations were very time consuming.

412. Strongly Disagree
413. Disagree
414. Neither Agree nor Disagree
415. Agree
416. Strongly Agree
417. Not Applicable

My app helped me to waste time less in order to exercise more.

418. Strongly Disagree
419. Disagree
420. Neither Agree nor Disagree
421. Agree
422. Strongly Agree

My app helped me feel that health outcomes are not out of my control.

423. Strongly Disagree
424. Disagree
425. Agree
426. Strongly Agree

My app helped me to enjoy exercise.

427. Strongly Disagree
428. Disagree
429. Agree
430. Strongly Agree

My app taught me how to exercise or operate the necessary equipment.

431. Strongly Disagree
432. Disagree
433. Agree
434. Strongly Agree
435. Not applicable

My app helped me to exercise without fear of injury.

436. Strongly Disagree
437. Disagree
438. Agree
439. Strongly Agree
440. Not applicable

My App helped me to find time to exercise or to exercise even though I didn't think I had the time.

441. Strongly disagree
442. Disagree
443. Agree
444. Strongly Agree
445. Lack of time is not an obstacle to exercising for me

My App helped me to complete my workout even though exercise normally makes me fatigued.

446. Strongly Disagree
447. Disagree
448. Agree
449. Strongly Agree
450. Getting fatigued is not an obstacle to exercising for me

My app helped me to complete my workout even though I have limited access to places where I can exercise.

451. Strongly Disagree
452. Disagree
453. Agree
454. Strongly Agree
455. Access to places where I can exercise is not an obstacle to exercising for me

My app helped me to overcome the embarrassment I feel when exercising.

456. Strongly Disagree
457. Disagree
458. Agree
459. Strongly Agree
460. Being embarrassed is not an obstacle to exercising for me

My app helped me to exercise without spending too much money.

461. Strongly Disagree
462. Disagree
463. Agree
464. Strongly Agree
465. Spending too much money is not an obstacle to exercising for me

My app helped me to exercise even though my friends and family did not support my effort.

466. Strongly Disagree
467. Disagree
468. Agree
469. Strongly Agree
470. Lack of support from friends and family is not an obstacle to exercising for me

My app helped me to exercise without taking too much time from my family responsibilities.

471. Strongly Disagree
472. Disagree
473. Agree
474. Strongly Agree
475. Taking too much time from family relationships is not an obstacle to exercising for me

My app helped me with my self-management skills, such as the ability to set personal goals, monitor my progress, or reward my progress toward such goals.

476. Strongly Disagree
477. Disagree
478. Agree
479. Strongly Agree

Please tell us what features of your app were most useful to you in helping you to increase exercise. Please note, we give bonuses for meaningful answers.

Please tell us whether any features of your app may have been disruptive, unhelpful, or may have inhibited your motivation to exercise. Please note, we give bonuses for meaningful answers.

The reason why I got my app was to help me exercise even though I did not feel I had the time.

480. Strongly Disagree
481. Disagree
482. Agree
483. Strongly Agree

The reason why I got my app was to help me address getting fatigued while exercising.

484. Strongly Disagree
485. Disagree
486. Agree
487. Strongly Agree

The reason why I got my app was to help me address my limited access to places where I can exercise.

488. Strongly Disagree
489. Disagree
490. Agree
491. Strongly Agree

The reason why I got my app was to help me address my feelings of embarrassment while exercising.

492. Strongly Disagree
493. Disagree
494. Agree
495. Strongly Agree

The reason why I got my app was to help me to exercise without spending too much money.

496. Strongly Disagree
497. Disagree
498. Agree
499. Strongly Agree

The reason why I got my app was to overcome the lack of support from friends and family.

500. Strongly Disagree
501. Disagree
502. Agree
503. Strongly Agree

The reason why I got my app was to find time to exercise despite my family responsibilities.

504. Strongly Disagree
505. Disagree
506. Agree
507. Strongly Agree

The reason why I got my app was to help me with my self-management skills, such as the ability to set personal goals, monitor my progress, or reward my progress toward such goals

508. Strongly Disagree
509. Disagree
510. Agree
511. Strongly Agree

IPAQ

Below is a list of things people might do while trying to increase or continue regular exercise. We are interested in exercises like running, swimming, brisk walking, bicycle riding, or aerobics classes. Whether you exercise or not, please rate how confident you are that you could really motivate yourself to do things like these consistently, for at least six months. Please select one number for each question. How sure are you that you can do these things?

How confident you are that you could really motivate yourself to do things like these consistently, for at least six months. Get up early, even on weekends, to exercise.

512. 1 - I know I cannot
513. 2
514. 3 - Maybe I can
515. 4
516. 5 - I know I can

Stick to your exercise program after a long, tiring day at work.

517. 1 -I know I cannot
518. 2
519. 3 - Maybe I can
520. 4
521. 5 - I know I can

Exercise even though you are feeling depressed.

522. 1 - I know I cannot
523. 2
524. 3 - Maybe I can
525. 4
526. 5 - I know I can

Set aside time for a physical activity program; that is, walking, jogging, swimming, biking, or other continuous activities for at least 30 minutes, 3 times per week.

527. 1 - I know I cannot
528. 2
529. 3 - Maybe I can
530. 4
531. 5 - I know I can

Continue to exercise with others even though they seem too fast or too slow for you.

532. 1 I know I cannot
533. 2
534. 3 - Maybe I can
535. 4
536. 5 - I know I can

Stick to your exercise program when undergoing a stressful life change (e.g., divorce, death in the family, moving).

537. 1 - I know I cannot
538. 2
539. 3 - Maybe I can
540. 4
541. 5 - I know I can

Attend a party only after exercising.

542. 1 - I know I cannot
543. 2
544. 3 - Maybe I can
545. 4
546. 5 - I know I can

Stick to your exercise program when your family is demanding more time from you.

547. 1 - I know I cannot
548. 2
549. 3 - Maybe I can
550. 4
551. 5 - I know I can

Stick to your exercise program when you have household chores to attend to.

552. 1 - I know I cannot
553. 2
554. 3 - Maybe I can
555. 4
556. 5 - I know I can

Stick to your exercise program even when you have excessive demands at work

557. 1 - I know I cannot
558. 2
559. 3 - Maybe I can
560. 4
561. 5 - I know I can

Stick to your exercise program when social obligations are very time consuming.

562. 1 - I know I cannot
563. 2
564. 3 - Maybe I can
565. 4
566. 5 - I know I can

Waste time less in order to exercise more.</div></div>

567. 1 - I know I cannot
568. 2
569. 3 - Maybe I can
570. 4
571. 5 - I know I can

We are interested in finding out about the kinds of physical activities that people do as part of their everyday lives. These questions will ask you about the time you spent being physically active in the last 7 days. Please answer each question even if you do not consider yourself to be an active person. Please think about the activities you do at work, as part of your house and yard work, to get from place to place, and in your spare time for recreation, exercise or sport. Think about all the vigorous and moderate activities that you did in the last 7 days. Vigorous physical activities refer to activities that take hard physical effort and make you breathe much harder than normal. Moderate activities refer to activities that take moderate physical effort and make you breathe somewhat harder than normal.

PART 1: JOB-RELATED PHYSICAL ACTIVITY The first section is about your work. This includes paid jobs, farming, volunteer work, course work, and any other unpaid work that you did outside your home. Do not include unpaid work you might do around your home, like housework, yard work, general maintenance, and caring for your family.

Do you currently have a job or do any unpaid work outside your home?

572. Yes
573. No

The next questions are about all the physical activity you did in the last 7 days as part of your paid or unpaid work. This does not include traveling to and from work.

During the last 7 days, on how many days did you do vigorous physical activities like heavy lifting, digging, heavy construction, or climbing up stairs as part of your work? Think about only those physical activities that you did for at least 10 minutes at a time.

574. 0
575. 1
576. 2
577. 3
578. 4
579. 5
580. 6
581. 7

For the next bunch of questions every time you are asked how much time you did something, please only use numbers and no letters to answer. E.G. if you exercised 1 hour and 30 minutes per day write 1 in the hour box and 30 in the minute box. Thank you

How much time did you usually spend on one of those days doing Vigorous physical activities as part of your work?

Again, think about only those physical activities that you did for at least 10 minutes at a time. During the last 7 days, on how many days did you do moderate physical activities like carrying light loads as part of your work? Please do not include walking.

582. 0
583. 1
584. 2
585. 3
586. 4
587. 5
588. 6
589. 7

How much time did you usually spend on one of those days doing Moderate physical activities as part of your work?

During the last 7 days, on how many days did you walk for at least 10 minutes at a time as part of your work? Please do not count any walking you did to travel to or from work.

590. 0
591. 1
592. 2
593. 3
594. 4
595. 5
596. 6
597. 7

How much time did you usually spend on one of those days walking as part of your work?

PART 2: TRANSPORTATION PHYSICAL ACTIVITY These questions are about how you traveled from place to place, including to places like work, stores, movies, and so on.

During the last 7 days, on how many days did you travel in a motor vehicle like a train, bus, car, or tram?

598. 0
599. 1
600. 2
601. 3
602. 4
603. 5
604. 6
605. 7

How much time did you usually spend on one of those days traveling in a train, bus, car, tram, or other kind of motor vehicle?

Now think only about the bicycling and walking you might have done to travel to and from work, to do errands, or to go from place to place.

During the last 7 days, on how many days did you bicycle for at least 10 minutes at a time to go from place to place?

606. 0
607. 1
608. 2
609. 3
610. 4
611. 5
612. 6
613. 7

How much time did you usually spend on one of those days to bicycle from place to place?

During the last 7 days, on how many days did you walk for at least 10 minutes at a time to go from place to place?

614. 0
615. 1
616. 2
617. 3
618. 4
619. 5
620. 6
621. 7

How much time did you usually spend on one of those days walking from place to place?

PART 3: HOUSEWORK, HOUSE MAINTENANCE, AND CARING FOR FAMILY. This section is about some of the physical activities you might have done in the last 7 days in and around your home, like housework, gardening, yard work, general maintenance work, and caring for your family.

Think about only those physical activities that you did for at least 10 minutes at a time. During the last 7 days, on how many days did you do vigorous physical activities like heavy lifting, chopping wood, shoveling snow, or digging in the garden or yard?

622. 0
623. 1
624. 2
625. 3
626. 4
627. 5
628. 6
629. 7

How much time did you usually spend on one of those days doing vigorous physical activities in the garden or yard?

Again, think about only those physical activities that you did for at least 10 minutes at a time. During the last 7 days, on how many days did you do moderate activities like carrying light loads, sweeping, washing windows, and raking in the garden or yard?

630. 0
631. 1
632. 2
633. 3
634. 4
635. 5
636. 6
637. 7

How much time did you usually spend on one of those days doing moderate physical activities in the garden or yard?

Once again, think about only those physical activities that you did for at least 10 minutes at a time. During the last 7 days, on how many days did you do moderate activities like carrying light loads, washing windows, scrubbing floors and sweeping inside your home?

638. 0
639. 1
640. 2
641. 3
642. 4
643. 5
644. 6
645. 7

How much time did you usually spend on one of those days doing moderate physical activities inside your home?

PART 4: RECREATION, SPORT, AND LEISURE-TIME PHYSICAL ACTIVITY. This section is about all the physical activities that you did in the last 7 days solely for recreation, sport, exercise or leisure. Please do not include any activities you have already mentioned.

Not counting any walking you have already mentioned, during the last 7 days, on how many days did you walk for at least 10 minutes at a time in your leisure time?

646. 0
647. 1
648. 2
649. 3
650. 4
651. 5
652. 6
653. 7

How much time did you usually spend on one of those days walking in your leisure time?

Think about only those physical activities that you did for at least 10 minutes at a time. During the last 7 day, on how many days did you do vigorous physical activities like aerobics, running, fast bicycling, or fast swimming in your leisure time?

654. 0
655. 1
656. 2
657. 3
658. 4
659. 5
660. 6
661. 7

How much time did you usually spend on one of those days doing vigorous physical activities in your leisure time?

Again, think about only those physical activities that you did for at least 10 minutes at a time. During the last 7 days, on how many days did you do moderate physical activities like bicycling at a regular pace, swimming at a regular pace, and doubles tennis in your leisure time?

662. 0
663. 1
664. 2
665. 3
666. 4
667. 5
668. 6
669. 7

How much time did you usually spend on one of those days doing moderate physical activities in your leisure time?

PART 5: TIME SPENT SITTING. The last questions are about the time you spend sitting while at work, at home, while doing course work and during leisure time. This may include time spent sitting at a desk, visiting friends, reading or sitting or lying down to watch television. Do not include any time spent sitting in a motor vehicle that you have already told me about.

During the last 7 days, how much time did you usually spend sitting on a weekday?

During the last 7 days, how much time did you usually spend sitting on a weekend day?

Attentional Check

Most favorite hobby. Hobbies are used by many people to explore their interests, help pass time and for entertainment. Social scientists have observed that some people may answer questions as quickly as possible without carefully reading the material. Please select that you do not have a hobby even if it is not true. This will verify your careful reading of this item.

670. Reading
671. Watching TV
672. Family Time
673. Fishing
674. Movies
675. Gardening
676. No Hobby
677. Walking
678. Exercise
679. Music
680. Other

Demographic Questions

What is your age?

What is your gender?

681. Male
682. Female

What is your current marital status?

683. Single
684. Married
685. Living in a marriage-like relationship
686. Divorced
687. Widowed

What is the highest degree or level of school you have completed? If currently enrolled, mark the previous grade or highest degree received.

688. No schooling completed
689. Nursery school to 8th grade
690. 9th, 10th or 11th grade
691. 12th grade, no diploma
692. High school graduate - high school diploma or the equivalent (for example: GED)
693. Some college credit, but less than 1 year
694. 1 or more years of college, no degree
695. Associate degree (for example: AA, AS)
696. Bachelor's degree (for example: BA, AB, BS)
697. Master's degree (for example: MA, MS, MEng, MEd, MSW, MBA)
698. Professional degree (for example: MD, DDS, DVM, LLB, JD)
699. Doctorate degree (for example: PhD, EdD)

What is your occupation?

What is your annual household income?

700. Less than $10,000
701. $10,000 to $19,999
702. $20,000 to $29,999
703. $30,000 to $39,999
704. $40,000 to $49,999
705. $50,000 to $59,999
706. $60,000 to $69,999
707. $70,000 to $79,999
708. $80,000 to $89,999
709. 90,000 to $99,999
710. $100,000 to $149,999
711. $150,000 or more

What is your height?

What is your current weight (lbs)? (numbers only please)

What is your ideal weight (lbs)? (numbers only please)

For demographic purposes only, which best describes your ethnic group?

712. White/Caucasian
713. Black/African American
714. American Indian/Alaska Native
715. Asian
716. Hawaiian Native
717. Pacific Islander
718. Other

Do you consider yourself Hispanic or Latino/Latina?

719. yes
720. No

Please briefly tell us about the study. Was the task easy; straightforward; hard; confusing; boring? Please feel free to share any other feedback about this task with us.

Thank you for taking part in our study! We very much appreciate your help!!!
